# In Vivo Ultrasound Characterization of the Tibial Nerve at the Supramalleolar Region

**DOI:** 10.1002/jfa2.70178

**Published:** 2026-07-15

**Authors:** María Benimeli‐Fenollar, Cecili Macián‐Romero, Lucía Carbonell‐José, María José Chiva‐Miralles, Enrique Sanchis‐Sales, Adrián Jordá‐Vallés, Vicent Tomás‐Martínez

**Affiliations:** ^1^ Department of Nursing University of Valencia Valencia Spain; ^2^ Independent Researcher Valencia Spain

**Keywords:** anatomical landmarks, ankle anatomy, neurovascular relationship, supramalleolar level, tibial nerve, ultrasound imaging, ultrasound‐guided procedures

## Abstract

**Background:**

Accurate knowledge of the supramalleolar anatomy of the tibial nerve is essential for safe ultrasound‐guided procedures and proximal nerve interventions in the foot and ankle. However, quantitative in vivo ultrasound reference data describing its positional anatomy at this level remain scarce.

**Methods:**

An in vivo ultrasound anatomical study was conducted in 100 healthy volunteers to evaluate the supramalleolar position of the tibial nerve. Measurements included the distance from the medial border of the tibial cortex to the center of the nerve, nerve depth from the skin surface, nerve perimeter, and the anatomical relationship between the tibial nerve and the posterior tibial artery. Associations with sex and anthropometric variables were analyzed using appropriate inferential statistical tests.

**Results:**

Ultrasound imaging demonstrated a stable and predictable anatomical configuration of the tibial nerve at the supramalleolar level, with bifurcation observed in only 1% of cases. Men showed a greater distance between the tibial nerve and the medial tibial cortex compared with women (*p* = 0.009), whereas women exhibited greater nerve depth (*p* = 0.003). The tibial nerve–tibial cortex distance was positively correlated with body weight (*p* < 0.001), body mass index (*p* = 0.001), and malleolar circumference (*p* = 0.004). No significant associations were observed between anthropometric variables and the anatomical relationship between the tibial nerve and the posterior tibial artery (*p* > 0.05).

**Conclusions:**

The tibial nerve at the supramalleolar level exhibits a stable in vivo anatomical arrangement with minimal branching variability. These ultrasound‐based reference values provide clinically relevant information that may enhance diagnostic assessment and support ultrasound‐guided procedural planning in the foot and ankle.

## 1 Introduction

Accurate localization of the tibial nerve (TN) is clinically relevant because it is routinely targeted in anesthetic procedures for foot surgery. Its relatively deep anatomical course and close spatial relationship with the posterior tibial artery (PTA) increase the technical complexity of tibial nerve interventions, particularly when procedures are performed using anatomical landmarks alone [1, 2, 3, 4, 5].

Accurate identification of its anatomical position is essential for diagnostic assessment, ultrasound‐guided procedures, and regional anesthesia at the ankle and distal leg. High‐resolution ultrasound has emerged as a valuable diagnostic tool for real‐time visualization of peripheral nerves, allowing precise assessment of nerve morphology, depth, and spatial relationships with surrounding structures.

Most anatomical knowledge regarding the distal course of the tibial nerve is derived from cadaveric studies. These investigations have primarily focused on describing the pattern and level of tibial nerve bifurcation and the origin of its terminal branches within the tarsal tunnel [6, 7, 8, 9, 10, 11, 12]. While these studies provide important anatomical insights, cadaveric findings may not accurately reflect the in vivo position of the nerve or its relationship with adjacent vascular and musculoskeletal structures during ultrasound examination [13].

To date, quantitative in vivo ultrasound data describing the supramalleolar position of the tibial nerve relative to fixed anatomical landmarks remain scarce. The lack of ultrasound‐based reference values at this level may limit diagnostic accuracy and procedural planning, particularly when ultrasound guidance is not immediately available or when anatomical variability is suspected. Moreover, the supramalleolar region represents a clinically relevant window, as the tibial nerve typically remains as a single trunk before distal branching occurs [7, 8, 9, 11, 14, 15]. This characteristic facilitates tibial nerve procedures and reduces the risk of incomplete nerve blockade [16, 17, 18].

Therefore, the aim of the present study was to provide an in vivo ultrasound‐based analysis of the tibial nerve at the supramalleolar level, describing its positional parameters, depth, perimeter, and anatomical relationship with the posterior tibial artery, as well as its variability according to sex and anthropometric characteristics. By establishing ultrasound‐based reference data, this study seeks to support diagnostic evaluation and ultrasound‐guided assessment of the tibial nerve in the distal leg and ankle.

## 2 Materials and Methods

### 2.1 Study Design and Participants

A cross‐sectional, descriptive ultrasound study was carried out at the University of Valencia to assess the anatomical location and morphometric features of the tibial nerve at the supramalleolar level. The study included 100 healthy adult volunteers (57 women and 43 men) aged between 18 and 75 years, who were recruited through public advertisements within the university community. No predefined sex allocation was established, and the final distribution reflected the availability of volunteers during the recruitment period. Sample size estimation was performed using the GRANMO sample size calculator (IMIM, Barcelona, Spain). A minimum sample of 97 participants was required to estimate the population mean with a 95% confidence level and a precision of ± 0.1 units, assuming a standard deviation of 0.5 units. No replacement losses were anticipated.

Eligibility criteria required the absence of lower limb neuropathies, prior ankle trauma or surgery, and systemic diseases known to affect peripheral nerves, such as diabetes mellitus or polyneuropathy. Participants were excluded if they presented with edema, varicose veins, or anatomical deformities that could interfere with ultrasonographic visualization of the tibial nerve (TN) or the posterior tibial artery (PTA).

All procedures were conducted in accordance with the ethical principles outlined in the Declaration of Helsinki and received approval from the Ethics Committee of the University of Valencia (approval code: H1477566491165). Written informed consent was obtained from all participants prior to enrollment.

### 2.2 Calibration

A calibration procedure was performed under the supervision of a clinician with extensive expertise in musculoskeletal ultrasound to ensure consistency of the measurement protocol. The calibration phase involved two examiners: the expert clinician and the principal investigator. Both independently identified the tibial nerve on transverse ultrasound images obtained 4 cm proximal to the inferior margin of the medial malleolus and performed all study measurements in a subgroup of 15 participants. Each examiner completed one full set of measurements for every participant during the calibration process. Individuals included in the calibration phase were excluded from the final study analysis.

Intraclass correlation coefficients demonstrated excellent reliability for the distance from the bony reference point to the center of the tibial nerve and for tibial nerve depth, with values of 0.956 (95% CI: 0.891–0.982) and 0.981 (95% CI: 0.952–0.993), respectively. In contrast, the intraclass correlation coefficient for tibial nerve perimeter showed moderate reliability, with a value of 0.547 (95% CI: 0.142–0.793). Agreement between the principal investigator and the expert clinician regarding the classification of the anatomical relationship between neurovascular structures was complete (100%), yielding a Kappa coefficient of 1, which corresponds to almost perfect agreement according to the Landis and Koch criteria.

### 2.3 Ultrasound Protocol

**FIGURE 1:**
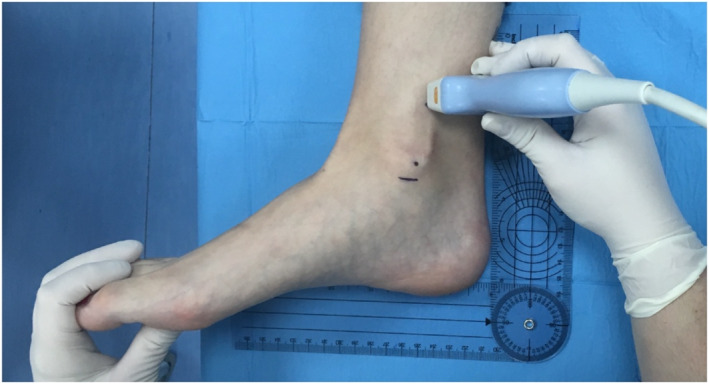
Identification and marking of the bony reference landmarks for the supramalleolar ultrasound assessment of the tibial nerve. The ultrasound transducer was positioned 4 cm proximal to the inferior border of the medial malleolus, with the ankle maintained in a neutral position.

Ultrasound examinations were performed by an experienced sonographer using a high‐resolution linear transducer. All measurements were obtained with participants in the supine position and the foot placed at a 90° angle relative to the tibia to standardize limb orientation. All measurements were obtained at a predefined supramalleolar level, corresponding to a transverse line located 4 cm proximal to the inferior border of the medial malleolus (Figure 1).

This anatomical level was selected for three main reasons: first, the medial border of the tibia can be reliably identified by palpation at this height; second, ultrasound visualization of the tibial nerve is facilitated due to reduced interference from superficial soft tissues compared with more proximal levels; and third, the likelihood of tibial nerve bifurcation into its terminal branches is minimal at this location.

**FIGURE 2:**
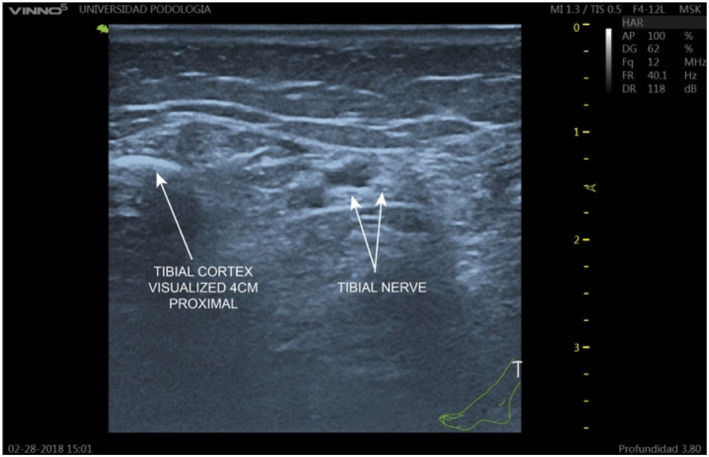
Ultrasound image of the tibial nerve (TN) at the supramalleolar level. The tibial nerve is identified as a predominantly hyperechoic structure with internal hypoechoic fascicles, displaying a characteristic honeycomb appearance, adjacent to the posterior tibial artery (PTA), which appears as a round anechoic structure. The tibial cortex, visualized 4 cm proximal to the inferior border of the medial malleolus, is seen as a curvilinear hyperechoic line, with its medial margin corresponding to the free cortical edge.

A high‐frequency linear transducer (4–12 MHz) (Vinno 5) (Vinno Technology Co., Suzhou, China) was positioned transversely along the medial aspect of the distal leg. The tibial nerve was identified as a hyperechoic, fascicular structure located next to the posterior tibial artery (Figure 2). Color Doppler imaging was used when necessary to confirm vascular structures and avoid misidentification.

Two orthogonal reference axes were defined to standardize measurements: a transverse axis (*X*‐axis) parallel to the skin surface and a vertical axis (*Y*‐axis) perpendicular to it. Linear distances and depth measurements were obtained using the ultrasound system's electronic calipers to ensure measurement accuracy.

To prevent confusion between the tibial nerve and adjacent tendinous structures, particularly the flexor hallucis longus tendon, plantar flexion of the hallux was performed during image acquisition when required.

**FIGURE 3:**
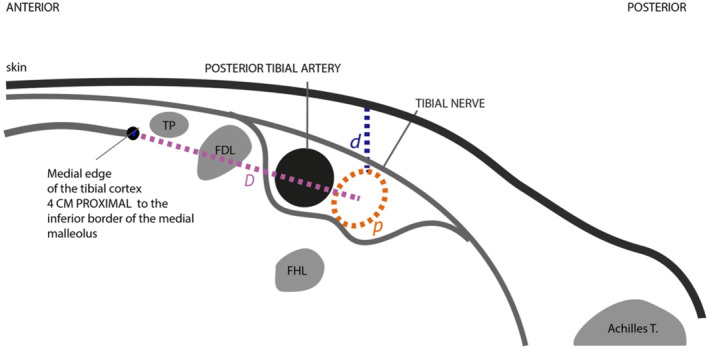
Schematic representation of a transverse ultrasound image of the neurovascular bundle at the supramalleolar level. From medial to lateral, the following structures are identified: posterior tibial tendon (PT), flexor digitorum longus tendon (FDL), posterior tibial artery, and tibial nerve. The flexor hallucis longus tendon (FHL) is in a deeper plane. Positional measurements include: **D**, the horizontal distance measured along the skin surface from the bony reference point to the center of the tibial nerve; **p**, the perimeter of the tibial nerve; and **d**, the vertical distance (depth) from the skin surface to the superior border of the tibial nerve perimeter. *Source:* authors' own work.

Measurements were obtained at the level of the bony reference point at the supramalleolar level (the medial edge of the tibial cortex 4 cm proximal to the inferior border of the medial malleolus) (Figure 3).

1. **Distance (D):** distance from the bony reference point to the center of the tibial nerve, measured in centimeters.
2. **Depth (d):** perpendicular distance from the skin surface to the upper edge of the tibial nerve perimeter.
3. **Perimeter (P):** measured along the outer contour of the tibial nerve. Tibial nerve perimeter was delineated to define the external contour of the nerve and to standardize assessment of its positional relationship with the posterior tibial artery within the neurovascular bundle. Perimeter measurements were used as anatomical reference parameters rather than as diagnostic morphometric variables.
4. **TN–PTA relationship:** classified according to the relative position of both structures.

Each measurement was performed three times by the same examiner, and the mean value was used for statistical analysis.

#### 2.3.1 Classification of Nerve—Artery Relationship

The anatomical relationship between the tibial nerve and the posterior tibial artery was classified according to a four‐type system based on their relative spatial arrangement. This classification scheme, originally proposed by Kim et al. [6], categorizes the nerve position as posterior, anterior, or lateral to the artery, as well as the presence of multiple nerve trunks.

**FIGURE 4:**
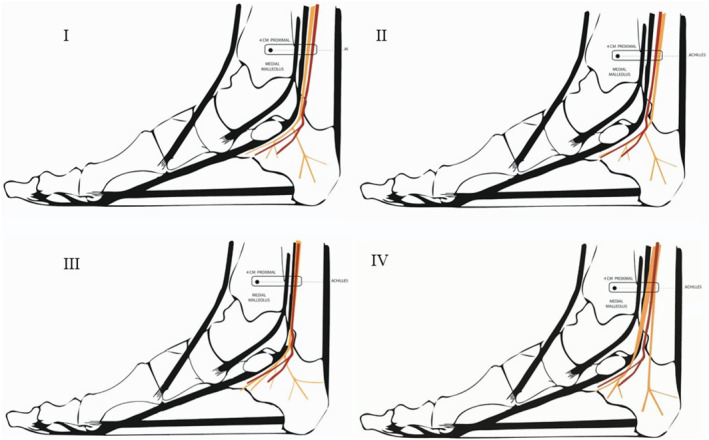
Schematic representation of the anatomical relationship patterns between the tibial nerve and the posterior tibial artery at the supramalleolar level, defined as 4 cm proximal to the inferior border of the medial malleolus. Four relationship types are illustrated: **Type I**, the tibial nerve is located posterior to the posterior tibial artery; **Type II**, the tibial nerve is positioned anterior to the artery; **Type III**, the tibial nerve lies deeper to the artery; and **Type IV**, the presence of tibial nerve bifurcation. *Source:* authors' original illustration.

In the present study, the tibial nerve was used as the reference structure for classification, allowing consistent identification of neurovascular patterns at the supramalleolar level (Figure 4). This approach has also been applied in previous anatomical investigations of the distal tibial nerve [13] and facilitates comparison across studies.

- **Type I:** After delineation of the tibial nerve perimeter, the nerve is located posterior to the posterior tibial artery.
- **Type II:** After delineation of the tibial nerve perimeter, the nerve is positioned anterior to the posterior tibial artery.
- **Type III:** After delineation of the tibial nerve perimeter, the nerve trunk lies deeper to the posterior tibial artery, beneath the vascular bundle.
- **Type IV:** The presence of more than one tibial nerve trunk, consistent with nerve bifurcation.

The distribution of these patterns was recorded for the total sample and by sex.

#### 2.3.2 Anthropometric Variables

Anthropometric data included: Age (years), Sex (male/female), Weight (kg), Height (cm), Body mass index (BMI, kg/m^2^) and Ankle circumference (cm). All measurements were collected by the same investigator to minimize inter‐observer variability.

### 2.4 Statistical Analysis

Statistical analyses were performed using IBM SPSS Statistics (version 22.0; IBM Corp., Armonk, NY, USA). Quantitative variables are reported as mean values with corresponding 95% confidence intervals (CI), while categorical variables are expressed as frequencies and percentages.

Group comparisons for continuous variables were conducted using Student's *t*‐test or one‐way analysis of variance (ANOVA), as appropriate. When applicable, post hoc comparisons were adjusted using the Bonferroni correction. Normality of data distribution was assumed based on the central limit theorem for sample sizes exceeding 30 participants.

Associations between categorical variables were evaluated using the Chi‐square test. Linear relationships between quantitative variables were assessed using Pearson's correlation coefficient. A two‐tailed *p*‐value < 0.05 was considered statistically significant.

## 3 Results

### 3.1 Participant Characteristics

A total of 100 healthy adult volunteers participated in the study, including 57 women and 43 men. The overall mean age of the sample was 29 years, with an age range from 19 to 74 years. The principal anthropometric characteristics of the study population are summarized in Table 1.

**TABLE 1:** Anthropometric characteristics of the study participants.

Variable	Mean (range)	95% CI
Age (years)	29.0 (19–74)	[26.6–31.4]
Height (m)	1.68 (1.51–1.93)	[1.66–1.70]
Weight (kg)	65.6 (40–105)	[62.9–68.2]
BMI (kg/m^2^)	23.1 (16.6–36.3)	[22.4–23.8]
Ankle circumference (cm)	24.9 (22–29.5)	[24.6–25.3]

### 3.2 Ultrasound Measurements of the Tibial Nerve

At the supramalleolar level, the tibial nerve was identified at a mean distance of 1.81 cm from the medial tibial cortex (bony reference point), with values ranging from 0.88 to 2.83 cm. The nerve was located at a mean depth of 1.24 cm beneath the skin surface (range: 0.60–2.59 cm). A summary of the ultrasound‐derived measurements of the tibial nerve is presented in Table 2.

**TABLE 2:** Ultrasound measurements of the tibial nerve (TN) in the study population.

Variable	MEAN(Range)	95% CI
TN‐tibial cortex distance at 4 cm (cm)	1.81 (0.88–2.83)	[1.75–1.89]
TN depth (cm)	1.24 (0.6–2.59)	[1.17–1.31]
TN perimeter (mm)	1.29 (0.8–1.77)	[1.25–1.36]

### 3.3 Tibial Nerve–Posterior Tibial Artery Anatomical Relationships

**FIGURE 5:**
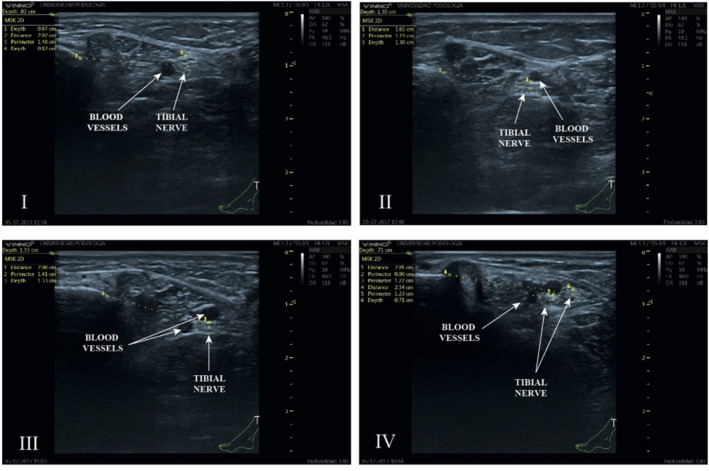
Ultrasound images illustrating the four anatomical relationship patterns between the tibial nerve (TN) and the posterior tibial artery (PTA) at the supramalleolar level. Type I, the tibial nerve is positioned posterior to the artery (61%); Type II, the tibial nerve lies anterior to the artery (9%); Type III, the tibial nerve is located deeper to the artery (29%); and Type IV, bifurcation of the tibial nerve (1%). White arrows indicate the position of the tibial nerve relative to the adjacent vascular structures.

Four distinct anatomical relationship patterns were identified at the supramalleolar level (Figure 5). The predominant configuration (61%) corresponded to a posterior position of the tibial nerve relative to the posterior tibial artery (Type I). Tibial nerve bifurcation was rarely observed, being identified in only one participant (1%), located 4 cm proximal to the inferior border of the medial malleolus.

### 3.4 Sex‐ and BMI‐Related Differences in Supramalleolar Tibial Nerve Position

**FIGURE 6:**
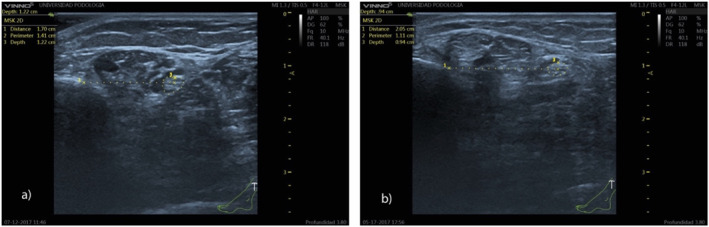
Ultrasound images illustrating the position of the tibial nerve at the supramalleolar level in women (a) and men (b). The distance from the bony reference point to the center of the tibial nerve is greater in men (b) (2.05 vs. 1.70 cm), whereas the nerve is located at a greater depth from the skin surface in women (a) (1.22 vs. 0.94 cm).

The influence of sex and BMI category on tibial nerve positional parameters is presented in Table 3. A significantly greater distance between the tibial nerve and the medial tibial cortex was observed in men compared with women (*p* < 0.01), whereas women exhibited a slightly greater nerve depth (*p* < 0.01) (Figure 6). With respect to BMI categories, significant differences were identified for the mediolateral distance from the tibial cortex to the center of the nerve, primarily between underweight and obese participants (*p* = 0.05). In contrast, tibial nerve depth did not differ significantly across BMI categories.

**TABLE 3:** Sex‐ and BMI‐related differences in supramalleolar tibial nerve positional parameters.

	N	Mean	95% CI	Statistical test
TN‐tibial cortex distance				
Sex				
Woman	57	1.74	[1.67–1.81]	Student t *p* value = 0.009*
Man	43	1.91	[1.80–2.03]	
BMI category				
1	5	1.56	[1.20–1.92]	ANOVA test *p* value = 0.025* Bonferroni post hoc 1 versus 4 *P* = 0.05
2	65	1.78	[1.71–1.85]	
3	27	1.90	[1.75–2.06]	
4	3	2.18	[1.59–2.77]	
TN depth				
Sex				
Woman	57	1.33	[1.23–1.43]	Student t *p* value = 0.003*
Man	43	1.12	[1.03–1.22]	
BMI category				
1	5	1.27	[0.98–1.57]	ANOVA test *p* value = 0.334
2	65	1.19	[1.10–1.28]	
3	27	1.30	[1.17–1.44]	
4	3	1.84	[1.26–2.42]	

*Note:* BMI category: 1 = Underweight; 2 = Normal weight; 3 = Overweight; 4 = Obesity.

^*^ Significant difference *p* < 0.05.

### 3.5 Sex‐ and BMI‐Related Variation in Supramalleolar Tibial Nerve–Artery Relationships

The distribution of anatomical relationship patterns between the tibial nerve and the posterior tibial artery according to sex and BMI category is presented in Supporting Information S1: Table S1. At the supramalleolar level, no statistically significant differences were detected in the position of the tibial nerve within the neurovascular bundle between men and women (*p* = 0.061). Similarly, the anatomical relationship patterns did not differ significantly across BMI categories (*p* = 0.802).

### 3.6 Influence of Anthropometric Variables on Supramalleolar Tibial Nerve–Artery Relationships

The analysis examining the anatomical relationship between the tibial nerve and the posterior tibial artery across different categories of weight, height, BMI, and malleolar circumference is presented in Supporting Information S1: Table S2. At the supramalleolar level, no statistically significant associations were identified between these anthropometric variables and the position of the tibial nerve within the neurovascular bundle, as assessed by one‐way ANOVA.

### 3.7 Correlation Analysis of Supramalleolar Tibial Nerve Positional Parameters

Supporting Information S1: Table S3 summarizes the Pearson correlation analyses performed among the quantitative variables included in the study. The mediolateral distance between the medial tibial cortex and the center of the tibial nerve showed a moderate positive correlation with body weight (*r* = 0.381) and body mass index (BMI) (*r* = 0.328).

Ankle (malleolar) circumference was also positively associated with the mediolateral position of the tibial nerve (*r* = 0.283), indicating that individuals with a larger malleolar perimeter tend to exhibit a greater distance between the tibial cortex and the nerve at the supramalleolar level. In addition, malleolar circumference demonstrated significant correlations with age (*r* = 0.331) and BMI (*r* = 0.537), reflecting its close relationship with overall body size parameters. Finally, tibial nerve depth increased in parallel with BMI, showing a positive linear correlation (*r* = 0.290).

## 4 Discussion

The present study provides quantitative in vivo ultrasound data describing the positional anatomy of the tibial nerve at the supramalleolar level, demonstrating a stable and predictable anatomical configuration in this region. High‐resolution ultrasound enabled consistent identification of the tibial nerve relative to fixed bony and vascular landmarks, supporting its value as a reliable diagnostic tool for nerve localization and assessment in the distal leg and ankle.

The initial hypothesis of the present study was that sex and anthropometric variables such as weight, height, BMI, and ankle circumference could influence the position of the tibial nerve and its anatomical relationship with the posterior tibial artery at the supramalleolar level. The results partially support this hypothesis, demonstrating that while several anthropometric factors affect the spatial position of the tibial nerve, they do not significantly modify its anatomical relationship with the posterior tibial artery at this level.

The anatomical reference used for all measurements was an imaginary horizontal line located 4 cm proximal to the inferior border of the medial malleolus, which was defined as the baseline from which all distances were calculated. This landmark was chosen first, because the medial border of the tibia is easily identifiable by palpation at this anatomical level. Moreover, this location ensures clear ultrasound identification of the tibial nerve. A more proximal level might have compromised nerve visualization due to increased subcutaneous tissue thickness. Finally, there is a lower probability that the tibial nerve has already bifurcated into its distal branches: the medial plantar nerve, the lateral plantar nerve, and the calcaneal nerve.

All measurements were obtained with the foot maintained at a 90° angle relative to the tibia to standardize the acquisition process and reduce potential measurement variability [6, 9, 12, 15, 19, 20]. Plantar flexion of the hallux was systematically performed to prevent misidentification of the flexor hallucis longus tendon as the tibial nerve on ultrasound imaging [21]. In line with previous studies [6, 9, 13, 22], two anatomical axes were defined to perform the measurements: a transverse *X*‐axis and a vertical *Y*‐axis.

The lack of universally accepted criteria for categorizing neurovascular relationships at the ankle has historically limited direct comparison between anatomical studies. In this investigation, the relationship between the tibial nerve and the posterior tibial artery was assessed using the four‐pattern classification originally described by Kim et al. [6], which defines neurovascular arrangements according to the relative position of the artery with respect to the nerve. For consistency, the tibial nerve was used as the reference structure.

At the supramalleolar level, the tibial nerve most commonly occupied a posterior position relative to the posterior tibial artery (Type I), accounting for 61% of cases. Notably, tibial nerve bifurcation was rarely observed at this level, occurring in only 1% of the study population. This finding is of particular relevance, as it suggests a high degree of anatomical uniformity before distal nerve branching. In contrast, cadaveric studies conducted by Kim et al. [6] reported a predominance of arterial positioning medial to the tibial nerve and a substantially higher frequency of nerve bifurcation. The markedly lower prevalence of branching observed in vivo at the supramalleolar level enhances anatomical predictability and facilitates ultrasound‐based nerve identification, potentially reducing uncertainty during diagnostic evaluation and procedural planning.

Sex‐related differences were also identified in tibial nerve positioning. Male participants demonstrated a greater horizontal distance between the tibial nerve and the medial tibial cortex, whereas female participants exhibited increased nerve depth. Despite these differences, the tibial nerve maintained a relatively superficial course at the supramalleolar level in both sexes. Variations in muscle mass, connective tissue distribution, and subcutaneous adipose tissue between men and women may account for these observations, as previously reported in anatomical and anthropometric literatura [23, 24, 25, 26].

Beyond sex‐related effects, several anthropometric parameters influenced the absolute spatial location of the tibial nerve. Body weight, body mass index, and ankle circumference were positively associated with the distance from the medial tibial cortex to the nerve center, while body mass index also correlated with increased nerve depth. These findings indicate that greater body mass and ankle morphology contribute to lateral displacement and deeper positioning of the tibial nerve. Importantly, however, these factors did not alter the relative anatomical relationship between the tibial nerve and the posterior tibial artery, underscoring the stability of the neurovascular configuration at the supramalleolar level across individuals with differing body composition.

The limited variability observed in the tibial nerve–posterior tibial artery relationship may be attributed to the more proximal anatomical location examined in this study. At the supramalleolar level, neurovascular structures are less constrained by the fibro‐osseous boundaries of the tarsal tunnel, which may contribute to a more consistent spatial arrangement [17]. From a diagnostic and interventional perspective, this predictability is particularly advantageous for ultrasound‐guided assessment, as it may reduce the likelihood of vascular misidentification and support safer and more reliable evaluation of the tibial nerve at this level.

Although the present investigation focuses on the supramalleolar region, previously published findings from the same research group describing the tibial nerve at the retromalleolar level offer relevant background information. That earlier work documented a higher frequency of tibial nerve bifurcation and greater anatomical variability, factors that may complicate nerve localization and procedural planning [13]. For this reason, a comparative overview of ultrasound findings obtained at both anatomical levels is provided as supplementary material (Supporting Information S1: Table S4), strictly for contextual purposes, while preserving the supramalleolar region as the primary subject of the current study.

From a clinical and diagnostic perspective, accurate knowledge of tibial nerve anatomy at the supramalleolar level is particularly important for ultrasound‐guided interventions, proximal nerve blocks, and surgical approaches involving the distal leg and ankle. At this level, the relative position of the tibial nerve and the posterior tibial artery was found to be largely consistent. Nevertheless, variations in nerve position related to sex and anthropometric characteristics emphasize the importance of individualized assessment to reduce the risk of neural or vascular injury.

With respect to clinical performance, landmark‐based supramalleolar approaches were associated with incomplete blocks in 14.5% of cases, a vascular puncture rate of 7.3%, and a peri‐ or intraneural puncture rate of 10.9%. The overall success rate achieved with conventional anatomical landmarks at this level was 78,2% [5], which is higher than that reported by some authors using retromalleolar ultrasound (66%) and landmark‐based techniques (22%) [21], but slightly lower (81,8%) than the success rate obtained in our previously published in vivo retromalleolar study using anatomically validated reference points [13]. These findings suggest that, despite the favorable anatomical configuration identified on ultrasound, accurate localization of the tibial nerve at the supramalleolar level may be technically more demanding when imaging guidance is not employed. The greater nerve depth at this level and the requirement for distal anesthetic diffusion to achieve complete sensory blockade may partly explain these results when surface landmarks alone are used. At present, there are no published clinical studies reporting success rates for landmark‐based tibial nerve blocks specifically performed at the supramalleolar level, which limits direct comparison with previous literature.

Collectively, these observations indicate that anatomical consistency does not necessarily translate into optimal clinical efficacy when landmark‐based techniques are applied. Consequently, the supramalleolar approach should be considered anatomically advantageous yet technically more demanding in the absence of real‐time imaging. Ultrasound guidance enables direct visualization of neural and vascular structures, supports accurate needle placement, and may help mitigate risks associated with variable tissue composition and increased nerve depth at this level. Although operator dependency remains a limitation of ultrasonography, particularly in musculoskeletal and peripheral nerve imaging, the implementation of standardized acquisition protocols and objective measurement criteria has been shown to improve reproducibility. Advances in imaging technology and operator training have further contributed to consistent and reliable ultrasound assessments across different clinical settings [27].

The relevance of proximal approaches to tibial nerve blockade has been emphasized in the literature for several decades. Early work by Gerbert demonstrated that variability in terminal branching of the posterior tibial nerve may compromise the effectiveness of distal nerve blocks, particularly when bifurcation occurs proximal to the injection site [14]. Subsequent clinical studies have explored more proximal tibial nerve block techniques using objective nerve localization methods, such as nerve stimulation. Doty Jr. et al. described the feasibility of a proximal tibial nerve block guided by neurostimulation [17], while Larrabure et al. evaluated a midleg approach in a large clinical series, reporting reliable nerve localization and a low incidence of vascular complications [16]. Although explicit success rates were not quantified in these studies, their findings support a consistent clinical rationale for proximal tibial nerve approaches aimed at reducing the impact of anatomical variability.

In this context, the present supramalleolar ultrasound study provides in vivo anatomical evidence supporting proximal strategies by demonstrating a low prevalence of tibial nerve bifurcation and a stable relationship between the tibial nerve and the posterior tibial artery.

To the best of our knowledge, this is the first study to provide in vivo ultrasound‐based reference values describing the supramalleolar position of the tibial nerve in relation to fixed bony landmarks. Previous anatomical investigations have largely relied on cadaveric material or focused on distal branching patterns, without quantifying the spatial relationship between the nerve and external anatomical references. By providing quantitative ultrasound data from a large cohort of healthy participants, the present study contributes novel anatomical information that may support safer and more accurate diagnostic and interventional procedures involving the tibial nerve.

These reference values should be interpreted as population‐based anatomical parameters rather than fixed coordinates. Whenever feasible, real‐time ultrasound guidance remains the most reliable approach for identifying neural and vascular structures prior to invasive procedures at the supramalleolar level.

### 4.1 Study Limitations

Several limitations should be acknowledged when interpreting the results of this study. Although the sample size was adequate, the participants were mainly young, healthy adults, which may restrict the applicability of the findings to older populations or to patients with peripheral nerve disorders, local anatomical deformities, edema, or inflammatory conditions. For this reason, the anatomical reference values presented here should be understood as population‐based descriptors rather than fixed parameters applicable to all clinical scenarios.

In addition, the present investigation was designed as an ultrasound‐based anatomical study and did not include a direct assessment of clinical or procedural outcomes. While this methodological approach is appropriate for defining in vivo anatomical reference data, future research combining ultrasonographic findings with clinical effectiveness and safety measures would help clarify the practical impact of supramalleolar tibial nerve assessment.

Another limitation is that measurement reliability was evaluated only at the intra‐observer level. Inter‐observer agreement was not assessed, and therefore the reproducibility of the protocol across examiners with different levels of ultrasound experience remains to be determined. Studies involving multiple independent observers would be valuable to confirm the robustness of the proposed measurements.

Another limitation of the present study relates to the morphometric assessment of the tibial nerve. Cross‐sectional area (CSA), which is currently considered the standard quantitative parameter in diagnostic peripheral nerve ultrasound, was not evaluated. Instead, tibial nerve perimeter was measured to delineate the external contour of the nerve and to standardize assessment of its positional relationship with the posterior tibial artery within the neurovascular bundle. In addition, perimeter measurements demonstrated lower inter‐examiner reliability compared with positional and depth measurements, likely reflecting the greater technical difficulty associated with consistent delineation of the nerve boundary on transverse ultrasound images. Therefore, perimeter values should be interpreted as anatomical reference parameters rather than diagnostic morphometric measurements.

Finally, despite the advantages of high‐resolution ultrasound for peripheral nerve imaging, the technique remains inherently operator dependent. Nonetheless, the implementation of standardized scanning protocols and objective measurement criteria may help minimize variability and promote consistent results across different clinical and research settings.

## 5 Conclusions

This ultrasound‐based study provides in vivo anatomical reference data describing the position of the tibial nerve at the supramalleolar level in relation to fixed bony landmarks and the posterior tibial artery. The supramalleolar region showed a relatively consistent neurovascular configuration, characterized by a low prevalence of tibial nerve bifurcation and a stable spatial relationship with the posterior tibial artery.

The tibial nerve was identified at a greater depth at this level, with positional variations related to sex and anthropometric characteristics, underscoring the importance of individualized anatomical assessment. Although the supramalleolar configuration appears anatomically favorable, clinical findings indicate that landmark‐based techniques at this level may remain technically demanding, particularly in the absence of imaging guidance.

Taken together, these findings highlight that anatomical predictability alone does not guarantee procedural efficacy or safety when surface anatomical references are used. Ultrasound suggests itself as a valuable tool to translate supramalleolar anatomical consistency into accurate nerve localization, improved procedural planning, and reduced risk of neurovascular complications.

The anatomical reference values reported in this study should be interpreted as population‐based parameters intended to support, but not replace, real‐time ultrasound guidance. Future studies integrating imaging findings with clinical outcomes are warranted to further define the role of supramalleolar ultrasound‐guided approaches in tibial nerve interventions.

## Author Contributions

**María Benimeli‐Fenollar:** conceptualization, methodology, validation, formal analysis, investigation, data curation, writing – original draft, writing – review and editing, visualization, project administration. **Cecili Macián‐Romero:** conceptualization, methodology, validation, resources, supervision, writing – review and editing. **Lucía Carbonell‐José:** conceptualization, writing – review and editing. **María José Chiva‐Miralles:** conceptualization, writing – review and editing. **Enrique Sanchis‐Sales:** conceptualization, writing – review and editing. **Adrián Jordá‐Vallés:** conceptualization, writing – review and editing. **Vicent Tomás‐Martínez:** conceptualization, writing – review and editing, visualization. All authors have read and agreed to the published version of the manuscript.

## Funding

The authors have nothing to report.

## Ethics Statement

The study was conducted in accordance with the Declaration of Helsinki, and approved by the Valencia University Ethics Committee (approval code: H1477566491165).

## Consent

Informed consent was obtained from all subjects involved in the study.

## Conflicts of Interest

The authors declare no conflicts of interest.

## Supporting information


Supporting Information S1

## Data Availability

The data that support the findings of this study are available from the corresponding author upon reasonable request.
