# Supplementary material for: In Vivo Ultrasound Characterization of the Tibial Nerve at the Supramalleolar Region
Source: J Foot Ankle Res. 2026 Jul 15;19(3):e70178. doi: 10.1002/jfa2.70178 (PMC13373442; doi:10.1002/jfa2.70178)
Supplement: Supplementary file 1 — Supporting Information S1 [file JFA2-19-e70178-s001.docx]

**Supplementary Table S1.** Supramalleolar tibial nerve–posterior tibial artery relationships stratified by sex and BMI category.

|  | **Type I N=61** | | **Type II N=9** | | **Type III N=29** | | **Type IV N=1** | | **Statistical Test** |
| --- | --- | --- | --- | --- | --- | --- | --- | --- | --- |
|  | ***N* (%)** | **95% IC** | ***N* (%)** | **95% IC** | ***N* (%)** | **95% IC** | ***N* (%)** | **95% IC** | **Chi^2^ Test** |
| Woman | 41 (71.9) | [59.2-81.9] | 4 (7.0) | [ 2.8-16.7] | 12 (21.1) | [12.5-33.3] | 0 (0.0) | [ 0-6.3] | P value=0.061 |
| Man | 20 (46.5) | [ 32.5-61.1] | 5 (11.6) | [5.1-24.4] | 17 (39.5) | [26.4-54.4] | 1 (2.3) | [0.4-12.1] |  |
| Cat. 1 | 3 (60.0) | [23.1-88.2] | 0 (0.0) | [0-43.4] | 2 (40.0) | [11.7-76.9] | 0 (0.0) | [ 0-43.4] | P value=0.802 |
| Cat. 2 | 42 (64.6) | [52.5-75.1] | 4 (6.2) | [2.4-14.8] | 18 (27.7) | [18.3-39.5] | 1 (1.5) | [18.3-39.6] |  |
| Cat. 3 | 14 (51.9) | [33.9- 69.3] | 5 (18.5) | [8.2-36.7] | 8 (29.6) | [ 15.9-48.4] | 0 (0.0) | [0-12.4] |  |
| Cat. 4 | 2 (66.7) | [20.7-93.8] | 0 (0.0) | [0-56.1] | 1 (33.3) | [6.1-79.2] | 0 (0.0) | [ 0-56.1] |  |

Type of anatomical relationship: type I: the TN is located posterior to the PTA; type II: the TN is located anterior to the PTA; type III: the TN is positioned deeper to the PTA; type IV: More than one nerve trunk corresponding to the TN is observed. BMI category: 1 = Underweight; 2 = Normal weight; 3 = Overweight; 4 = Obesity.

*Significant difference *p* < 0.05

**Supplementary Table S2.** Association of anthropometric variables with supramalleolar tibial nerve–posterior tibial artery relationships.

|  | **Type I**  ***N*=61** | | **Type II**  ***N*=9** | | **Type III**  ***N*=29** | | **Type IV**  ***N*=1** | | **ANOVA Test** |
| --- | --- | --- | --- | --- | --- | --- | --- | --- | --- |
|  | **Mean** | **95% IC** | **Mean** | **95% IC** | **Mean** | **95% IC** | **Mean** | **95% IC** |  |
| Weight | 64.1 | [ 60.7-67.4] | 68.2 | [ 59.1-77.3] | 68.1 | [ 62.7-73.4] | 62.0 | - | P value=0.524 |
| Height | 1.67 | [ 1.65-1.70] | 1.68 | [1.59-1.77] | 1.70 | [ 1.67-1.73] | 1.75 | - | P value=0.519 |
| BMI | 22.8 | [22.0-23.6] | 24.1 | [ 21.6-26.7] | 23.4 | [ 21.9-24.9] | 20.2 | - | P value=0.542 |
| Malleolar circumference | 24.6 | [24.2-25.1] | 25.6 | [24.3-26.8] | 25.5 | [24.8-26.2] | 26.0 | - | P value=0.116 |

Type of anatomical relationship: type I: the TN is located posterior to the PTA; type II: the TN is located anterior to the PTA; type III: the TN is positioned deeper to the PTA; type IV: More than one nerve trunk corresponding to the TN is observed.

*Significant difference *p* < 0.05

**Supplementary Table S3. Pearson correlations between supramalleolar tibial nerve positional parameters and anthropometric variables (N = 100).**

|  | | TN  perimeter | TN  depth | Malleolar circumference | Age | BMI | Height | Weight |
| --- | --- | --- | --- | --- | --- | --- | --- | --- |
| TN-tibial cortex distance | Pearson Correlation (two-tailed-sig.) | 0.134  0.184 | -0.103  0.306 | 0.283**  0.004 | 0.118  0.243 | 0.328**  0.001 | 0.246*  0.014 | 0.381**  0.000 |
| TN perimeter | Pearson Correlation (two-tailed-sig.) |  | 0.114  0.258 | 0.133  0.188 | 0.200*  0.046 | 0.152  0.132 | 0.085  0.399 | 0.181  0.071 |
| TN depth | Pearson Correlation (two-tailed-sig.) |  |  | 0.108  0.285 | 0.181  0.072 | 0.290**  0.003 | -0.235* 0.018 | 0.071  0.484 |
| Malleolar circumference | Pearson Correlation (two-tailed-sig.) |  |  |  | 0.331**  0.001 | 0.537**  0.000 | 0.628**  0.000 | 0.750**  0.000 |
| Age | Pearson Correlation (two-tailed-sig.) |  |  |  |  | 0.442**  0.000 | -0.166  0.099 | 0.215*  0.032 |
| BMI | Pearson Correlation (two-tailed-sig.) |  |  |  |  |  | 0.136  0.176 | 0.812**  0.000 |
| Height | Pearson Correlation (two-tailed-sig.) |  |  |  |  |  |  | 0.684**  0.000 |

TN=tibial nerve

**p*<0,05; ***p*<0,01

**Supplementary Table S4.** Summary of clinical outcomes reported for supramalleolar and retromalleolar tibial nerve block approaches.

| **Variable** | **Retromalleolar level** | **Supramalleolar level** | **Main comparative interpretation** |
| --- | --- | --- | --- |
| **TN – tibial cortex distance** | Greater distance in men vs. women (*p* < 0.001) | Greater distance in men vs. women (*p* = 0.009) | Sex consistently influences mediolateral TN position at both  levels. |
| **TN depth** | Greater depth in women (*p* = 0.047) | Greater depth in women (*p* = 0.003) | Sex-related depth differences persist proximally and distally. |
| **BMI categories vs. TN position** | No significant differences | Significant differences in TN–cortex distance (*p* =  0.025; underweight vs. obesity) | BMI influences TN position only at the supramalleolar level. |
| **TN - PTA relationship vs. sex** | Significant differences (women mainly Type I; men more Types III–IV)  (*p* < 0.001) | No significant differences (*p* = 0.061) | Sex affects neurovascular configuration retromalleolarly but not supramalleolarly. |
| **TN - PTA relationship vs. weight** | Significant differences, mainly Type I vs. Type III  (*p* = 0.004) | No significant differences | Weight modifies TN–PTA arrangement only at the  retromalleolar level. |
| **TN - PTA relationship vs. height** | Significant differences, mainly Type I vs. Type III  (*p* < 0.001) | No significant differences | Height influences TN–PTA relationship distally, not proximally. |
| **TN - PTA relationship vs. ankle**  **circumference** | Significant differences, mainly Type I vs. Type IV  (*p* = 0.006) | No significant differences | Ankle morphology affects distal neurovascular anatomy only. |
| **TN distance and**  **weight** | Positive correlation (*p* =  0.001) | Positive correlation (*p* <  0.001) | Weight consistently associated with  increased TN distance at both levels. |
| **TN distance and**  **height** | Positive correlation (*p* <  0.001) | Not significant | Height influences TN position mainly  at the retromalleolar level. |
| **TN distance and BMI** | Not significant | Positive correlation (*p* = 0.001) | BMI effect emerges proximally rather than distally. |
| **TN distance and ankle circumference** | Positive correlation (*p* = 0.002) | Positive correlation (*p* = 0.004) | Ankle circumference consistently reflects TN mediolateral  displacement. |
| **TN depth and BMI** | Not significant | Positive correlation (*p* = 0.003) | BMI-related depth changes observed only supramalleolarly. |
| **TN distance and BMI** | Not significant | Positive correlation (*p* = 0.001) | BMI effect emerges proximally rather than distally. |
| **TN distance and ankle circumference** | Positive correlation (*p* = 0.002) | Positive correlation (*p* = 0.004) | Ankle circumference consistently reflects TN mediolateral  displacement. |
| **TN depth and BMI** | Not significant | Positive correlation (*p* = 0.003) | BMI-related depth changes observed only supramalleolarly. |

**Supplementary Table S4** summarizes the statistically significant associations identified at the retromalleolar and supramalleolar levels. Retromalleolar data were previously published by the authors [13] and are included here exclusively for comparative and contextual purposes. Only results with p < 0.05 are reported. Type of anatomical relationship: type I, the tibial nerve (TN) is located posterior to the posterior tibial artery (PTA); type II, the TN is located anterior to the PTA; type III, the TN is positioned deeper to the PTA; type IV, more than one nerve trunk corresponding to the TN is observed.
